# A novel *Drosophila* SOD2 mutant demonstrates a role for mitochondrial ROS in neurodevelopment and disease

**DOI:** 10.1002/brb3.73

**Published:** 2012-06-25

**Authors:** Alicia M Celotto, Zhaohui Liu, Andrew P VanDemark, Michael J Palladino

**Affiliations:** 1 Department of Pharmacology and Chemical Biology, University of Pittsburgh School of MedicinePittsburgh, Pennsylvania, 15261; 2 Pittsburgh Institute for Neurodegenerative Diseases, University of Pittsburgh School of MedicinePittsburgh, Pennsylvania, 15261; 3 Department of Biological Sciences, University of PittsburghPittsburgh, Pennsylvania, 15260

**Keywords:** *Drosophila melanogaster*, MnSOD, motoneuron axonal targeting, roGFP, ROS, SOD2

## Abstract

Reactive oxygen species (ROS) play essential roles in cell signaling, survival, and homeostasis. Aberrant ROS lead to disease and contribute to the aging process. Numerous enzymes and vigilant antioxidant pathways are required to regulate ROS for normal cellular health. Mitochondria are a major source of ROS, and mechanisms to prevent elevated ROS during oxidative phosphorylation require super oxide dismutase (SOD) activity. SOD2, also known as MnSOD, is targeted to mitochondria and is instrumental in regulating ROS by conversion of superoxides to hydrogen peroxide, which is further broken down into H_2_O and oxygen. Here, we describe the identification of a novel mutation within the mitochondrial SOD2 enzyme in *Drosophila* that results in adults with an extremely shortened life span, sensitivity to hyperoxia, and neuropathology. Additional studies demonstrate that this novel mutant, *SOD2*^*bewildered*^, exhibits abnormal brain morphology, suggesting a critical role for this protein in neurodevelopment. We investigated the basis of this neurodevelopmental defect and discovered an increase in aberrant axonal that could underlie the aberrant neurodevelopment and brain morphology defects. This novel allele, *SOD2*^*bewildered*^, provides a unique opportunity to study the effects of increased mitochondrial ROS on neural development, axonal targeting, and neural cell degeneration in vivo.

## Introduction

Reactive oxygen species (ROS) are produced at the highest concentrations within the mitochondria and consist of superoxide anion (O_2_^−^•), hydrogen peroxide (H_2_O_2_), and hydroxyl radicals (OH•). ROS are a normal byproduct of mitochondrial oxidative phosphorylation and are kept in check by cytosolic and mitochondrial antioxidant enzymes. It is known that ROS play an important role in regulating cell death and differentiation, suggesting their levels need to be tightly regulated for normal development, particularly within the brain (Finkel 2003; Ikonomidou and Kaindl 2011). ROS also serve critical signaling roles; hence, their levels must be tightly regulated to avoid cellular damage and dysfunction, particularly within mitochondria.

Mitochondrial ROS has been implicated in numerous diseases and aging, including many degenerative diseases, such as Parkinson's, Alzheimer's, and amyotrophic lateral sclerosis diseases, as well as normal and premature aging (Wallace 1992). Human mutations in *SOD2* are thought to play a role in numerous human disease conditions including cancer, mitochondrial disease, cardiopathy, diabetic neuropathy, and neurodegeneration (Rosenblum et al. 1996; Valenti et al. 2004; Mollsten et al. 2007). Within the human *SOD2* gene six mutations have been characterized: three mutations have been identified within promoter region that presumably reduce expression (Xu et al. 1999, 2007, 2008), one mutation affects the mitochondrial targeting of the enzyme (Rosenblum et al. 1996), and two missense mutations affect coding exon 3 (Borgstahl et al. 1996; Hernandez-Saavedra and McCord 2003). SOD2^Ala16Val^ affects the MTS and is associated with cardiomyopathy (Rosenblum et al. 1996; Valenti et al. 2004) and diabetic nephropathy (Mollsten et al. 2007). There has been significant work performed in model systems to understand the role of SOD2. In a murine model, mice lacking SOD2 (*SOD2*^*tm1Cje*^) develop dilated cardiomyopathy and neonatal lethality (Li et al. 1995); this same mutation in a different genetic background exhibits inhibition or inactivation of electron transport chain and other mitochondrial enzymes, and results in the accumulation of oxidative DNA damage (Melov et al. 1999). In *Drosophila*, previous studies have shown that *SOD2* RNAi and null mutations are associated with reduced longevity and neural dysfunction (Kirby et al. 2002; Duttaroy et al. 2003; Belton et al. 2006; Martin et al. 2009). Here, we report a novel missense mutation affecting SOD2 in *Drosophila* that leads to reduced longevity, sensitivity to hyperoxia, progressive increased mitochondrial ROS accumulation, neurodegeneration, and abnormal brain morphology. Our data demonstrate aberrant axonal targeting that likely underlies the abnormal brain morphology. Importantly, in silico studies support the conclusion that this mutation does not result in a major structural change to the SOD2 protein, yet dramatic reductions in steady state protein levels result, suggesting a marked increase in protein turnover of this mutant mitochondrial protein.

## Materials and Methods

### Fly husbandry, life spans, and stress-sensitivity tests

Flies were maintained on standard cornmeal, molasses food. Life spans and stress-sensitivity tests were performed at 25 and 29°C, as previously reported (Palladino et al. 2002, 2003; Celotto et al. 2006b; Fergestad et al. 2006b, 2008; Seigle et al. 2008). The *SOD2* mutant reported here was initially studied in the lab of Dr. Barry Ganetzky at the University of Wisconsin Madison where it was known as “hr2” and was identified in our previous screen of conditional mutants (Palladino et al. 2002). The *SOD2* deficiency line utilized is Df(2R)Exel7145 and was obtained from the Bloomington Stock Center.

### Western blot

Four fly heads were homogenized in 60 μL ice-cold lysis buffer (50 mmol/L Tris pH 6.8, 10% glycerol, 2% SDS, 0.01% bromophenol blue) in the presence of a protease inhibitor cocktail (1 mmol/L PMSF, 0.5 μg/mL Pepstatin A, 1 μg/mL Leupeptin). The homogenates were sonicated for 10 min and centrifuged at 10,000 RCF for 10 min. Samples were boiled for 5 min and loaded on a 12% SDS–polyacrylamide gel, then blotted onto a 0.2 μm PVDF membrane. The membrane was blocked with 1% milk/PBST for 1 h and then incubated with anti-SOD2 (1:2500; LSbio B3694) and anti-TPI (1:1000; Protein Tech, chicago, Illinois) antibodies overnight at 4°C. Membranes were washed with PBST and incubated with secondary antibody (1:4000, HRP goat antirabbit) for 1 h at room temperature. Membranes were washed with PBST and treated with ECL reagent (Thermo Scientific 32106, Waltham, Massachusetts) for 1 min. The membranes were immediately exposed to film and developed. Band densities were analyzed using Image J software (NIH).

### Modeling of SOD2 protein

A homology model of a *Drosophila* SOD2 monomer (with and without the G138D substitution) was generated via the program MUSTER (Wu and Zhang 2008) using *Caenorhabditis elegans* MnSOD2 (3dc6) as a structural template. Refinement of the resulting homology model was performed using ModRefiner (Xu and Zhang 2011) or a fragment guided MD simulation FG-MD (Zhang et al. 2011), and did not yield any significant alterations. Similar results were also obtained using MODELLER. The position of monomers within the SOD2 tetramer was determined by structural alignment to the *C. elegans* tetramer. The positions of manganese and hydroxyl ions were inferred from their positions within the *C. elegans* structures. The resulting distances between these ions and their hydrogen bonding partners are unchanged in this model.

### Hyperoxia sensitivity assays

*SOD2*^*bwd*^*/CyO* animals were mated to *Df7145/CyO* animals. Eggs were laid and 20 1st instar larvae were transferred to 10 separate vials. Vials were covered in cheesecloth, placed in a sealed container continuously infused with 20%, 40%, or 100% oxygen (balanced with nitrogen). Once animals eclosed the vials were removed from container, genotyped, and analyzed.

### Ratiometric analysis of ROS levels in adult brains

MTSroGFP2 analysis was performed as previously published (Liu et al. 2012). In summary, whole brains of 1 day or 3 days old adult animals were dissected in PBS. Genotypes used were: *elav-Gal4; UASB-MTSroGFP2 SOD2*^*bewildered*^*/Df7145* (mutant) and *elav-Gal4; UASB-MTSroGFP2 SOD2*^*bewildered*^*/CyO* (heterozygote) and *elav-Gal4; UASB-MTSroGFP2* (control). After dissection, brains were placed in mounting medium (Vectashield; Vector H-1000, Vector Laboratories, Burlingame, California) on a cover slip. Olympus confocal FV1000 microscope equipped with lasers for 405 and 488 nm excitation was used for imaging. Images were collected with a 20× lens in multi-track mode with line switching between 488 nm excitation and 405 nm excitation. The MTSroGFP2 emission fluorescence was collected with a 510–540 nm emission band-pass filter. *Z*-scan by 10 μm was used to achieve a whole brain image. Eight regions per brain were analyzed from 5 to 6 independent brains per genotype.

### Hematoxylin and eosin histology

Using previously published methods, *Drosophila* brains were dissected, fixed, paraffin embedded, and stained using hematoxylin (cell bodies) and eosin (neuropil) (Palladino et al. 2002; Celotto et al. 2006a). Standard light microscopy and a digital camera were used to document brain pathologies.

### FasII staining for ectopic motoneuron targeting analysis

Third instar larvae were dissected in PBS to expose the bodywall muscles and ventral ganglion without disruption of motoneuron and neuromuscular junctions (Jarecki and Keshishian 1995). Larvae were fixed in 4% paraformaldehyde for 20 min, washed three times for 10 min with PBT (0.1% Triton-×100 in PBS), and incubated with PBTB blocking solution (0.1% BSA in PBT) for 2 h at room temperature or 4°C overnight (Brent et al. 2009). Larvae were then incubated in Fas II primary antibody (University of California) at 1:10 in PBTB overnight at 4°C, and washed with PBT (three times for 10 min) (Hummel et al. 2000). Finally, larvae were incubated in Alexa 633 goat antimouse IgG secondary antibody (Invitrogen, Grand Island, New York) at 1:450 in PBTB for 1 h at room temperature, washed with PBT (three times for 10 min), and mounted in mounting medium (Vectashield; Vector H-1000) on glass microscope slide covered with cover slip. An Olympus FV1000 confocal microscope equipped with a 633 nm laser was used for imaging. Images were collected with a 600 to 700 nm emission band-pass filter under a 40× objective.

### Statistical analyses

Longevity assays were analyzed by log-rank. Chi-square test was used to determine statistical significance of the hyperoxia assays, *P < 0.05. The redox data were analyzed by PRISM software using a student's *t*-test, *P < 0.05, ***P < 0.001. For the targeting assay, statistical analysis was performed by PRISM software using a one-way ANOVA, *P < 0.05, **P < 0.01.

## Results

### Identification of a novel SOD2 missense mutation

**Figure 1:**
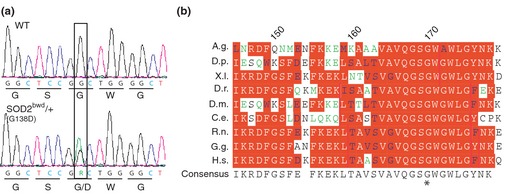
Identification of a novel mutation in *Drosophila* SOD2. (a) Sequence chromatographs of wildtype (WT-top) and SOD2^bwd^/+ (bottom) showing the G to A transition (boxed) resulting in a glycine to aspartic acid substitution. (b) Protein alignment illustrating the location of the SOD2 mutation (*) and demonstrating that it is located within an extremely conserved section of the protein. (A.g.: *Anopheles gambiae*, D.p.: *Drosophila pseudoobscura*, X.l.: *Xenopus laevis*, D.r.: *Danio rerio*, D.m.: *Drosophila melanogaster*, C.e.: *Caenorhabditis elegans*, R.n.: *Rattus norvegicus*, G.g.: *Gallus gallus*, H.s.: *Homo sapiens*). Numbers above sequence correlate with consensus sequence.

Previous studies of conditional locomotor mutants in *Drosophila* have identified novel mutations in key proteins involved in ion homeostasis, bioenergetics, neural signaling, synaptic transmission, and neuromuscular function (Siddiqi and Benzer 1976; Littleton et al. 1993; Palladino et al. 2002, 2003; Celotto et al. 2006a,b; Fergestad et al. 2006b). We identified an extraordinarily “bang-sensitive” autosomal recessive mutant that paralyzes conditionally upon exposure to mechanical stress. We positionally cloned the affected gene, which fails to complement deficiency Df7145 (Parks et al. 2004). As Df7145 is a deletion affecting many genes, we sequenced candidates within the interval and identified a novel missense mutation affecting an extremely conserved portion of the SOD2 protein. The mutation, named *SOD2 bewildered (bwd)*, is a G to A transition affecting amino acid 138 of the fly SOD2 protein resulting in a glycine (G) to an aspartic acid (D) (Fig. 1a). *SOD2*^*bwd*^ represents a novel mutant, the first missense mutation of *SOD2* described in this model system, and affects a highly conserved region of the SOD2 protein (Fig. 1b). Homozygous *SOD2*^*bwd*^ animals exhibit less than Mendelian expected viability; in matings between heterozygotes ∼5% of F1 animals rather than the expected 1/3 are homozygous.

### SOD2^bwd^ mutants have a severely reduced life span

**Figure 2:**
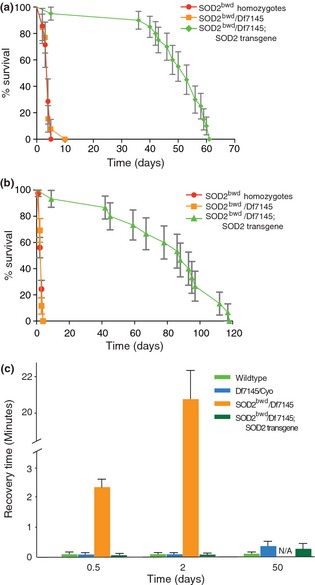
Life span analysis and rescue of SOD2 deficiency. (a) Life span of *SOD2*^*bwd*^ homozygotes (red), *SOD2*^*bwd*^*/Df7145* (orange), and *SOD2*^*bwd*^*/Df7145; SOD2 transgene* (green) at 29°C. (b) Life span of *SOD2*^*bwd*^ homozygotes (red), *SOD2*^*bwd*^*/Df7145* (orange), and *SOD2*^*bwd*^*/Df7145; SOD2 transgene* (green) at 25°C. The inclusion of a *SOD2*^*+*^ genomic transgene rescues the *SOD2*^*bwd*^*/Df7145* longevity defect to normal wildtype life span. (c) Stress-sensitive locomotor paralysis was examined and found to be strikingly progressive. *SOD2*^*bwd*^*/Df7145* animals (orange bars) soon after eclosion remain paralyzed for ∼2 min, whereas paralysis lasted over 20 min on day 2. Wildtype, heterozygous Df7145 and transgenically rescued animals do not exhibit paralysis on days 0.5 and 2. Heterozygous *Df7145* and rescued animals begin to show a modest paralysis phenotype on day 50. N/A reflects the fact that SOD2^bwd^/*Df7145* animals are not alive at that time.

We examined longevity of *SOD2*^*bwd*^ and it was found to be markedly reduced relative to wildtype flies. The typical life span of *Drosophila* is temperature dependent; however, *SOD2*^*bwd*^ animals do not live much longer than 5 days at either 25 or 29°C (Fig. 2a and b, red and orange lines). The longevity defect can be transgenically rescued with a described *SOD2* genomic transgene (Mockett et al. 1999), which was observed at 25 and 29°C (Fig. 2a and b, green lines). This transgene can also fully rescue the stress-induced locomotor paralysis seen in *SOD2*^*bwd*^ animals (Fig. 2c). These data are consistent with *SOD2*^*bwd*^ being a recessive mutation responsible for both the observed longevity and locomotor phenotypes.

### Altered stability rather than structure underlie SOD2^bwd^ pathogenesis

**Figure 3:**
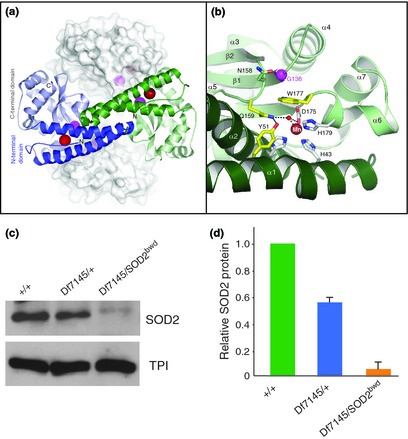
SOD2 structure and location of G138D. (a) Homology model of the fly SOD2 tetramer generated using MUSTER. The blue and green subunits are shown as a backbone cartoon and the positions of the N- and C-terminal domains are indicated. The location of the Mn ion within the model is indicated by a red sphere, whereas the Cα atom of residue G138 is indicated by a magenta sphere. Two additional subunits are shown as a transparent surface in white. (b) Ribbon diagram of the region surrounding G138 in our model. The green subunit has been rotated nearly 180° from panel a. In this view, residues that coordinate the Mn ion are shown as white sticks, whereas residues critical for substrate access and enzymatic activity are indicated by yellow sticks. The position of the coordinated hydroxyl is indicated. Hydrogen bonds within the active site coordinated by Q159 are indicated by dotted lines. (c) Western blot analysis reveals reduce steady state SOD 2 protein in SOD2^bwd^ mutant animals compared with the wildtype control (+/+). TPI (triose phosphate isomerase) was used as the loading control. (d) The relative ratios of the SOD2 protein were calculated using Image J (NIH) using three separate blots. The heterozygous SOD2 deficiency (*Df7145/+)* is at 72.4% of normal, and *SOD2*^*bwd*^*/Df7145* animals have 6.14% of normal SOD2 protein.

To further understand the effect the *SOD2*^*bwd*^ missense mutation (G138D) might have upon the SOD2 protein, we utilized in silico analyses. We generated a *Drosophila* SOD2 homology model using the program MUSTER (Wu and Zhang 2008) and the structure of the *C. elegans* manganese superoxide dismutase (3DC6) (Trinh et al. 2008) as the structural template (64% identical: Fig. 3a). The fold consists of N- and C-terminal domains with the catalytically important manganese ion residing between them. Conserved residues critical for ion coordination and enzymatic activity are therefore found within both domains. These include Trp-177, which forms a side of the SOD2 active site cavity. Mutations resulting in either alanine or phenylalanine substitutions at this position are known to reduce the catalytic rate over 100-fold in the human enzyme (Cabelli et al. 1999). Similarly, residues corresponding to *Drosophila* SOD2 Q159 and Y51 form a hydrogen bond network leading into the active site in human SOD2. A Tyr to Phe substitution at this position leads to a significant decrease in catalytic active without a decrease in stability or any substantial structural changes (Greenleaf et al. 2004). These findings for the human enzyme support the notion that even small perturbations in the active site may produce profound effects on enzymatic activity.

The two most likely explanations for the effect of a G138D substitution within SOD2 are that the G138D substitution is affecting catalysis directly through a change in the position of important active site residues or that decreased function of SOD2 is the result of decreased protein stability. We note that in our homology model, Gly138 is positioned near the surface of the C-terminal domain adjacent to residues that form the active site pocket, notably Trp-177. In addition, its position is also adjacent to the loop connecting β2 and α5, which contains Gln-159. Therefore, the *SOD2*^*bwd*^ mutant may result in subtle effects within either the active site pocket and/or the hydrogen bonding network within the active site. Despite being nonconservative, a G138D substitution would not appear to present any obvious packing or structural defects within the context of our homology model. As Gly138 is positioned at the end of a short loop, the decrease in backbone flexibility caused by a G138D mutant may cause changes in protein stability or protein folding.

The apparent lack of a striking structural change by the mutation is in stark contrast to the phenotypes seen in *SOD2*^*bwd*^ animals. The mutation is fully recessive, and thus would be predicted to be a loss-of-function mutant. This led us to investigate whether the basis of pathogenesis might be altered protein stability. Western blot analysis has demonstrated a significant decrease in the level of SOD2 protein within the heterozygous deficiency (*Df7145/+*) as well as *SOD2*^*bwd*^ animals (Fig. 3c and d). These data demonstrate that *SOD2*^*bwd*^ exhibit ∼6% of normal steady state protein levels, which is consistent with the interpretation that this is a strong loss-of-function mutation.

### *SOD2*^*bwd*^ mutants are sensitive to hyperoxia

**Figure 4:**
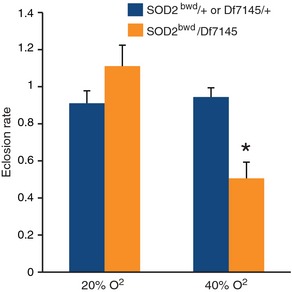
*SOD2*^*bwd*^ mutants are sensitive to hyperoxia. Eclosion rates were measured in animals raised at normoxia (20% O_2_) and hyperoxia (40% O_2_), and a significant reduction in survival is seen in SOD2^bwd^/Df7145 transheterozygotes (orange) compared with sibling controls (blue). 100% O_2_ was also tested; however, neither control nor mutant animals survived to adulthood.

Mitochondrial respiration is one of the key producers of superoxide within the cell, likely explaining why a mitochondrial targeted SOD is necessary for cellular health and animal survival. Most animals are able to exist at a range of effective oxygen concentrations; however, those with severe mitochondrial dysfunction can be hypersensitive to elevations in oxygen or hyperoxia (Jamieson et al. 1986). Mice deficient in SOD2 exhibit reduced survival rates that are inversely proportional to the percentage of oxygen in the air (Asikainen et al. 2002). We investigated whether *SOD2*^*bwd*^ flies were similarly sensitive to hyperoxia. Eclosion assays were performed to determine the percentage of adults eclosing from known numbers of embryoes under conditions of normoxia and hyperoxia. In these assays, *SOD2*^*bwd*^ animals show a decreased survival rate relative to wildtype control animals but only under conditions of hyperoxia (Fig. 4).

### Mitochondrial ROS are increased in *SOD2* ^*bwd*^ mutants

**Figure 5:**
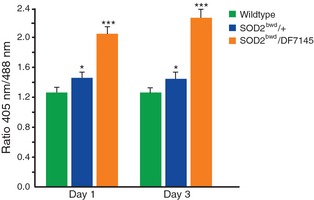
An increase in ROS is seen in *SOD2*^*bwd*^ mutant brains. The level of ROS as measured by the fluorescent ratio of MTSroGFP2 at 405 nm (oxidized) and 488 nm (reduced) demonstrates a significant increase in both *SOD2*^*bwd*^*/+* (blue) and *SOD2*^*bwd*^*/Df7145* (orange) brains compared with wildtype (green) brains on days 1 and 3.

The *SOD2*^*bwd*^ mutant is a strong loss-of-function, and thus mitochondrial antioxidant properties are predicted to be severely compromised. We utilized a recently developed genetically encoded mitochondrial redox sensor to measure ROS within these mutants (Liu et al. 2012). Transgenic animals bearing *UASB-MTSroGFP2* expressed MTS roGFP2 within the nervous system using *elavGAL4* and were used to measure the mitochondrial redox potential with ratiometric confocal microscopy (Liu et al. 2012). Mitochondrial ROS is markedly increased, even in very young *SOD2*^*bwd*^*/Df7145* adults compared with wildtype (Fig. 5). Although *SOD2*^*bwd*^ is phenotypically recessive, a modest but significant increase in mitochondrial redox potential is observed in heterozygotes (Fig. 5).

### Aberrant brain morphology and neuropathology in *SOD2* ^*bwd*^ mutants

**Figure 6:**
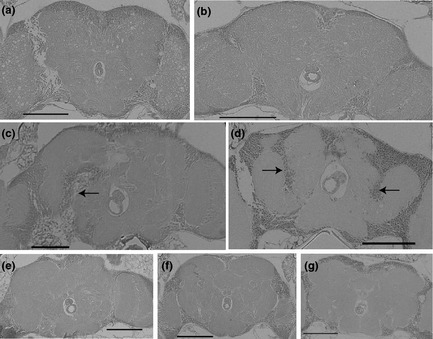
Adult abnormal brain morphology and neuropathology in *SOD2*^*bwd*^ mutants. (a and b) Vacuolar pathology was observed throughout the central brain of *SOD2*^*bwd*^*/Df7145* animals reared at 29°C (a) and at 25°C (b). (c) Abnormal localization of cell bodies in the neuropil of the central brain of SOD2^bwd^/Df7145 transheterozygotes at 22°C on day 2. Arrow points to the area of central brain infiltrated by aberrant cell bodies. (d) Abnormal localization of cell bodies in the neuropil of the central brain of SOD2^bwd^ homozygotes at 22°C on day 1.5. Again arrows identify aberrant cell bodies. (e) Df7145/+ heterozygotes at 22°C on day 14 show a reduced presence of spongiform degeneration. (f) SOD2^bwd^/+ heterozygotes at 22°C on day 14 show a reduced presence of spongiform degeneration. (g) Transgenic rescue of SOD2^bwd^/Df7145 brains at 22°C on day 20 demonstrate the complete reversal of spongiform degeneration. Scale bars are 100 μm.

Previous studies have shown an enrichment in mutants with conditional locomotor dysfunction and those with neurodegeneration (Palladino et al. 2002). Furthermore, elevated ROS and mitochondrial dysfunction have both been extensively associated with various neurodegenerative conditions (Lebovitz et al. 1996; Paul et al. 2007). These findings prompted us to examine whether *SOD2*^*bwd*^ animals exhibit neuropathology. We discovered extensive neurodegeneration throughout the brain of *SOD2*^*bwd*^ flies but not in those also bearing the genomic *SOD2* transgene (Figs. 6a, b, e, and f compared with 6g). Surprisingly, we discovered brains with clusters of nuclei located within the central neuropile. The neuropile of the central brain typically has peripheral clusters of nuclei and only sporadic nuclei inside the neuropile (Fig. 6c and d compared with 6g). The presence of large clusters of nuclei within the neuropile is highly abnormal and was never observed in wildtype, heterozygote, or transgenic rescue control animals (Fig. 6).

### Aberrant axonal targeting in *SOD2* ^*bwd*^ mutants

**Figure 7:**
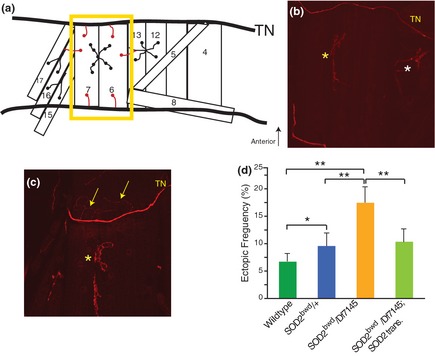
Increase in ectopic targeting of motoneurons in *SOD2*^*bwd*^ mutants. (a) Diagram describing layout of bodywall muscles and motoneurons in the third instar larvae. Muscles are labeled by number. TN is the transverse nerve. The yellow box identifies muscles 6 and 7 that were used for the assessment of ectopic motoneuron targeting. Normal neuromuscular junctions are drawn in black, whereas red are ectopic muscles 6 and 7 motoneurons. (b) FasII staining of wildtype TN and NMJ showing muscles 6, 7, 12, and 13. Yellow asterisk identifies the normal NMJ to muscles 6 and 7. White asterisk identifies the normal NMJ to muscles 12 and 13. (c) FasII staining of *SOD2*^*bwd*^*/Df7145* mutant. Yellow asterisk identifies the normal NMJ to muscles 6 and 7. Numerous ectopic neural projections are shown (yellow arrows). (d) Quantification of ectopic frequency seen in wildtype (green), *SOD2*^*bwd*^*/*^*+*^ heterozygotes (blue), *SOD2*^*bwd*^*/Df7145* animals (orange), and transgenic SOD2 rescue animals (light green).

The aberrant morphology of *SOD2*^*bwd*^ brains with large clusters of internally localized cell bodies within the neuropile suggests that SOD2 function is required for normal neurodevelopment. We investigated whether the defect might arise from aberrant axonal targeting. Although such assays have not been demonstrated in the fly CNS, assays of motoneuron targeting in the PNS have been previously described (Jarecki and Keshishian 1995). Using these assays we identified a significant increase in the frequency of ectopic motoneurons within *SOD2*^*bwd*^*/Df7145* mutants, consistent with an axonal targeting defect. Importantly, this ectopic outgrowth phenotype is rescued with the transgenic *SOD2* construct (Fig. 7). In agreement with our data demonstrating that a modest increase in mitochondrial ROS in *SOD2*^*bwd*^*/*^*+*^ heterozygotes (Fig. 5), we see a modest but significant increase in the frequency of ectopic neuronal targeting in *SOD2* heterozygous animals as well (Fig. 7).

## Discussion

SOD2 proteins perform a critical function in antioxidant defense within mitochondria. This function is required for health and viability with age, especially within the nervous system, and loss of this function is known to be deleterious. Using a forward genetic approach we positionally cloned and identified a novel pathogenic mutation affecting SOD2 in *Drosophila*. This novel mutant, *SOD2*^*bwd*^, exhibits phenotypes known to be associated with SOD2 dysfunction, including reduced longevity and neurodegeneration. However, our studies of *SOD2*^*bwd*^ demonstrate that this mutant results in reduced steady state protein levels, stress-sensitive paralysis, neurodevelopmental defects, neuropathology, and aberrant axonal targeting not previously associated with SOD2 dysfunction.

Surprisingly, modeling of the missense mutant does not predict an alteration in the structure of the SOD2 protein despite an amino acid substitution (G138D) that is different in both size and charge. These structural studies employed multiple protein modeling algorithms, which failed to produce a structure with significant alterations. These findings are from in silico studies and it remains possible that a structure derived by other methods, such as from a protein crystal, may identify changes that were not observed by these methods. Nonetheless, these studies suggested that the mutant protein could retain wildtype function and that altered stability of the protein might underlie *SOD2*^*bwd*^ pathogenesis. Consistent with this conclusion, steady state SOD2 protein levels were examined by Western blot and revealed a significant reduction from that of controls. These studies suggest that the mutant protein is unstable; however, additional experiments (e.g., pulse chase studies) to directly measure protein stability will be needed to verify that the reduced protein levels are the result of altered protein stability. Cytosolic proteins that are unstable can be the result of chaperone-mediated targeting to the proteasome, as is the case for a missense mutation affecting the TPI protein (Hrizo and Palladino 2010). As SOD2 is made in the cytosol, but is localized to the mitochondrial matrix, it will be important to determine how and where the mutant protein is detected and whether a similar mechanism regulates stability of this class of protein.

Stress-sensitive paralysis is a unique conditional locomotor phenotype that has been studied in flies for four decades (Benzer 1971; Wu and Ganetzky 1980; Ganetzky and Wu 1982; Pavlidis and Tanouye 1995; Palladino et al. 2002, 2003; Zhang et al. 2002; Tan et al. 2004; Hekmat-Scafe et al. 2006). Several such stress- or bang-sensitive (BS) mutants have been isolated and cloned and many have been found to affect cellular energetics (Pavlidis et al. 1994; Celotto et al. 2006b; Fergestad et al. 2006a). Several have been shown to specifically result from altered mitochondrial function (Royden et al. 1987; Zhang et al. 1999; Celotto et al. 2006a; Fergestad et al. 2006a). Intriguingly, numerous BS mutants are the result of altered neural excitability, have been shown to exhibit a seizure phenotype, and have been shown to model epilepsy in flies (Pavlidis and Tanouye 1995; Kuebler and Tanouye 2000, 2002; Reynolds et al. 2004; Tan et al. 2004; Parker et al. 2011). The finding of a strong loss-of-function allele of SOD2 with stress-sensitive paralysis demonstrates that SOD2 function is required for normal neural signaling and locomotor function. Further studies will be required to determine whether *SOD2*^*bwd*^ mutants exhibit convulsive seizures and reduced seizures thresholds akin to numerous other stress-sensitive mutants.

The finding of aberrant brain morphology in *SOD2*^*bwd*^ mutants is a novel phenotype to be associated with SOD2 dysfunction. This neuroanatomical defect observed in the adult brain suggested massive dysfunction in neurodevelopment of this important structure that could result from a general and widespread axonal targeting defect. Aberrant axonal targeting was confirmed using an established assay at the NMJ where aberrant targeting events can be quantified. These data support the conclusion that aberrant axonal targeting likely underlies the aberrant brain morphology observed. Although these are novel phenotypes associated with SOD2 dysfunction, they are supported by recent work which has identified a link between synaptic outgrowth and oxidative stress resulting from modulation of the JNK/AP-1 pathway in *Drosophila* (Milton et al. 2011). Others have demonstrated a connection between ROS and neural development resulting from hyperoxia and increase in neurite outgrowth (Katoh et al. 1997) and that proper ROS levels are required for proper neurogenesis (Suzukawa et al. 2000) and normal growth cone formation and neurite outgrowth (Munnamalai and Suter 2009). *SOD2*^*bwd*^ mutants represent a tractable model system to investigate the mechanism by which altered mitochondrial ROS result in aberrant neurodevelopment, and further studies will be needed to fully understand the genesis of the altered neuroanatomy.

## References

[b1] Asikainen TM, Huang TT, Taskinen E, Levonen AL, Carlson E, Lapatto R (2002). Increased sensitivity of homozygous Sod2 mutant mice to oxygen toxicity. Free Radic. Biol. Med.

[b2] Belton A, Paul A, Duttaroy A (2006). Deletions encompassing the manganese superoxide dismutase gene in the Drosophila melanogaster genome. Genome.

[b3] Benzer S (1971). From the gene to behavior. JAMA.

[b4] Borgstahl GE, Parge HE, Hickey MJ, Johnson MJ, Boissinot M, Hallewell RA (1996). Human mitochondrial manganese superoxide dismutase polymorphic variant Ile58Thr reduces activity by destabilizing the tetrameric interface. Biochemistry.

[b5] Brent J, Werner K, McCabe BD (2009). Drosophila larval NMJ immunohistochemistry. J. Vis. Exp.

[b6] Cabelli DE, Guan Y, Leveque V, Hearn AS, Tainer JA, Nick HS (1999). Role of tryptophan 161 in catalysis by human manganese superoxide dismutase. Biochemistry.

[b7] Celotto AM, Frank AC, McGrath SW, Fergestad T, Van Voorhies WA, Buttle KF (2006a). Mitochondrial encephalomyopathy in Drosophila. J. Neurosci.

[b8] Celotto AM, Frank AC, Seigle JL, Palladino MJ (2006b). Drosophila model of human inherited triosephosphate isomerase deficiency glycolytic enzymopathy. Genetics.

[b9] Duttaroy A, Paul A, Kundu M, Belton A (2003). A Sod2 null mutation confers severely reduced adult life span in Drosophila. Genetics.

[b10] Fergestad T, Bostwick B, Ganetzky B (2006a). Metabolic disruption in Drosophila bang-sensitive seizure mutants. Genetics.

[b11] Fergestad T, Ganetzky B, Palladino MJ (2006b). Neuropathology in Drosophila membrane excitability mutants. Genetics.

[b12] Fergestad T, Olson L, Patel KP, Miller R, Palladino MJ, Ganetzky B (2008). Neuropathology in Drosophila mutants with increased seizure susceptibility. Genetics.

[b13] Finkel T (2003). Oxidant signals and oxidative stress. Curr. Opin. Cell Biol.

[b14] Ganetzky B, Wu CF (1982). Indirect suppression involving behavioral mutants with altered nerve excitability in *Drosophila melanogaster*. Genetics.

[b15] Greenleaf WB, Perry JJ, Hearn AS, Cabelli DE, Lepock JR, Stroupe ME (2004). Role of hydrogen bonding in the active site of human manganese superoxide dismutase. Biochemistry.

[b16] Hekmat-Scafe DS, Lundy MY, Ranga R, Tanouye MA (2006). Mutations in the K+/Cl− cotransporter gene kazachoc (kcc) increase seizure susceptibility in Drosophila. J. Neurosci.

[b17] Hernandez-Saavedra D, McCord JM (2003). Paradoxical effects of thiol reagents on Jurkat cells and a new thiol-sensitive mutant form of human mitochondrial superoxide dismutase. Cancer Res.

[b18] Hrizo SL, Palladino MJ (2010). Hsp70- and Hsp90-mediated proteasomal degradation underlies TPI sugarkill pathogenesis in Drosophila. Neurobiol. Dis.

[b19] Hummel T, Krukkert K, Roos J, Davis G, Klambt C (2000). Drosophila Futsch/22C10 is a MAP1B-like protein required for dendritic and axonal development. Neuron.

[b20] Ikonomidou C, Kaindl AM (2011). Neuronal death and oxidative stress in the developing brain. Antioxid. Redox Signal.

[b21] Jamieson D, Chance B, Cadenas E, Boveris A (1986). The relation of free radical production to hyperoxia. Annu. Rev. Physiol.

[b22] Jarecki J, Keshishian H (1995). Role of neural activity during synaptogenesis in Drosophila. J. Neurosci.

[b23] Katoh S, Mitsui Y, Kitani K, Suzuki T (1997). Hyperoxia induces the differentiated neuronal phenotype of PC12 cells by producing reactive oxygen species. Biochem. Biophys. Res. Commun.

[b24] Kirby K, Hu J, Hilliker AJ, Phillips JP (2002). RNA interference-mediated silencing of Sod2 in Drosophila leads to early adult-onset mortality and elevated endogenous oxidative stress. Proc. Natl. Acad. Sci. USA.

[b25] Kuebler D, Tanouye MA (2000). Modifications of seizure susceptibility in Drosophila. J. Neurophysiol.

[b26] Kuebler D, Tanouye M (2002). Anticonvulsant valproate reduces seizure-susceptibility in mutant Drosophila. Brain Res.

[b27] Lebovitz RM, Zhang H, Vogel H, Cartwright J, Dionne L, Lu N (1996). Neurodegeneration, myocardial injury, and perinatal death in mitochondrial superoxide dismutase-deficient mice. Proc. Natl. Acad. Sci. USA.

[b28] Li Y, Huang TT, Carlson EJ, Melov S, Ursell PC, Olson JL (1995). Dilated cardiomyopathy and neonatal lethality in mutant mice lacking manganese superoxide dismutase. Nat. Genet.

[b29] Littleton JT, Bellen HJ, Perin MS (1993). Expression of synaptotagmin in Drosophila reveals transport and localization of synaptic vesicles to the synapse. Development.

[b30] Liu Z, Celotto AM, Romero G, Wipf P, Palladino MJ (2012). Genetically encoded redox sensor identifies the role of ROS in degenerative and mitochondrial disease pathogenesis. Neurobiol. Dis.

[b31] Martin I, Jones MA, Rhodenizer D, Zheng J, Warrick JM, Seroude L (2009). Sod2 knockdown in the musculature has whole-organism consequences in Drosophila. Free Radic. Biol. Med.

[b32] Melov S, Coskun P, Patel M, Tuinstra R, Cottrell B, Jun AS (1999). Mitochondrial disease in superoxide dismutase 2 mutant mice. Proc. Natl. Acad. Sci. USA.

[b33] Milton VJ, Jarrett HE, Gowers K, Chalak S, Briggs L, Robinson IM (2011). Oxidative stress induces overgrowth of the Drosophila neuromuscular junction. Proc. Natl. Acad. Sci. USA.

[b34] Mockett RJ, Orr WC, Rahmandar JJ, Benes JJ, Radyuk SN, Klichko VI (1999). Overexpression of Mn-containing superoxide dismutase in transgenic *Drosophila melanogaster*. Arch. Biochem. Biophys.

[b35] Mollsten A, Marklund SL, Wessman M, Svensson M, Forsblom C, Parkkonen M (2007). A functional polymorphism in the manganese superoxide dismutase gene and diabetic nephropathy. Diabetes.

[b36] Munnamalai V, Suter DM (2009). Reactive oxygen species regulate F-actin dynamics in neuronal growth cones and neurite outgrowth. J. Neurochem.

[b37] Palladino MJ, Hadley TJ, Ganetzky B (2002). Temperature-sensitive paralytic mutants are enriched for those causing neurodegeneration in Drosophila. Genetics.

[b38] Palladino MJ, Bower JE, Kreber R, Ganetzky B (2003). Neural dysfunction and neurodegeneration in Drosophila Na+/K+ ATPase alpha subunit mutants. J. Neurosci.

[b39] Parker L, Padilla M, Du Y, Dong K, Tanouye MA (2011). Drosophila as a model for epilepsy: bss is a gain-of-function mutation in the para sodium channel gene that leads to seizures. Genetics.

[b40] Parks AL, Cook KR, Belvin M, Dompe NA, Fawcett R, Huppert K (2004). Systematic generation of high-resolution deletion coverage of the *Drosophila melanogaster* genome. Nat. Genet.

[b41] Paul A, Belton A, Nag S, Martin I, Grotewiel MS, Duttaroy A (2007). Reduced mitochondrial SOD displays mortality characteristics reminiscent of natural aging. Mech. Ageing Dev.

[b42] Pavlidis P, Tanouye MA (1995). Seizures and failures in the giant fiber pathway of Drosophila bang-sensitive paralytic mutants. J. Neurosci.

[b43] Pavlidis P, Ramaswami M, Tanouye MA (1994). The Drosophila easily shocked gene: a mutation in a phospholipid synthetic pathway causes seizure, neuronal failure, and paralysis. Cell.

[b44] Reynolds ER, Stauffer EA, Feeney L, Rojahn E, Jacobs B, McKeever C (2004). Treatment with the antiepileptic drugs phenytoin and gabapentin ameliorates seizure and paralysis of Drosophila bang-sensitive mutants. J. Neurobiol.

[b45] Rosenblum JS, Gilula NB, Lerner RA (1996). On signal sequence polymorphisms and diseases of distribution. Proc. Natl. Acad. Sci. USA.

[b46] Royden CS, Pirrotta V, Jan LY (1987). The tko locus, site of a behavioral mutation in *D. melanogaster*, codes for a protein homologous to prokaryotic ribosomal protein S12. Cell.

[b47] Seigle JL, Celotto AM, Palladino MJ (2008). Degradation of functional triose phosphate isomerase protein underlies sugarkill pathology. Genetics.

[b48] Siddiqi O, Benzer S (1976). Neurophysiological defects in temperature-sensitive paralytic mutants of *Drosophila melanogaster*. Proc. Natl. Acad. Sci. USA.

[b49] Suzukawa K, Miura K, Mitsushita J, Resau J, Hirose K, Crystal R (2000). Nerve growth factor-induced neuronal differentiation requires generation of Rac1-regulated reactive oxygen species. J. Biol. Chem.

[b50] Tan JS, Lin F, Tanouye MA (2004). Potassium bromide, an anticonvulsant, is effective at alleviating seizures in the Drosophila bang-sensitive mutant bang senseless. Brain Res.

[b51] Trinh CH, Hunter T, Stewart EE, Phillips SE, Hunter GJ (2008). Purification, crystallization and X-ray structures of the two manganese superoxide dismutases from *Caenorhabditis elegans*. Acta Crystallogr. Sect. F Struct. Biol. Cryst. Commun.

[b52] Valenti L, Conte D, Piperno A, Dongiovanni P, Fracanzani AL, Fraquelli M (2004). The mitochondrial superoxide dismutase A16V polymorphism in the cardiomyopathy associated with hereditary haemochromatosis. J. Med. Genet.

[b53] Wallace DC (1992). Diseases of the mitochondrial DNA. Annu. Rev. Biochem.

[b54] Wu CF, Ganetzky B (1980). Genetic alteration of nerve membrane excitability in temperature-sensitive paralytic mutants of *Drosophila melanogaster*. Nature.

[b55] Wu S, Zhang Y (2008). MUSTER: Improving protein sequence profile-profile alignments by using multiple sources of structure information. Proteins.

[b56] Xu D, Zhang Y (2011). Improving the physical realism and structural accuracy of protein models by a two-step atomic-level energy minimization. Biophys. J.

[b57] Xu Y, Krishnan A, Wan XS, Majima H, Yeh CC, Ludewig G (1999). Mutations in the promoter reveal a cause for the reduced expression of the human manganese superoxide dismutase gene in cancer cells. Oncogene.

[b58] Xu Y, Fang F, Dhar SK, St Clair WH, Kasarskis EJ, St Clair DK (2007). The role of a single-stranded nucleotide loop in transcriptional regulation of the human sod2 gene. J. Biol. Chem.

[b59] Xu Y, Fang F, Dhar SK, Bosch A, St Clair WH, Kasarskis EJ (2008). Mutations in the SOD2 promoter reveal a molecular basis for an activating protein 2-dependent dysregulation of manganese superoxide dismutase expression in cancer cells. Mol. Cancer Res.

[b60] Zhang YQ, Roote J, Brogna S, Davis AW, Barbash DA, Nash D (1999). Stress sensitive B encodes an adenine nucleotide translocase in Drosophila melanogaster. Genetics.

[b61] Zhang H, Tan J, Reynolds E, Kuebler D, Faulhaber S, Tanouye M (2002). The Drosophila slamdance gene: a mutation in an aminopeptidase can cause seizure, paralysis and neuronal failure. Genetics.

[b62] Zhang J, Liang Y, Zhang Y (2011). Atomic-level protein structure refinement using fragment-guided molecular dynamics conformation sampling. Structure.

